# Comparison of the effects of two extraction methods on the alveolar ridge preservation of Maxillary Anterior Teeth

**DOI:** 10.12669/pjms.39.2.6643

**Published:** 2023

**Authors:** Si Jiang, Biao Zhou, Zheng Li, Juan Gao, Pei Wang

**Affiliations:** 1 Si Jiang, Department of Stomatology, Baoding First Central Hospital, Baoding 071000, Hebei, P. R. China; 2 Biao Zhou, Department of Stomatology, The No.2 Hospital of Baoding, Baoding, Hebei 071000, P. R. China; 3 Zheng Li, Department of Stomatology, The No.2 Hospital of Baoding, Baoding, Hebei 071000, P. R. China; 4 Juan Gao, Department of Stomatology, The No.2 Hospital of Baoding, Baoding, Hebei 071000, P. R. China; 5 Pei Wang, Department of Stomatology, The No.2 Hospital of Baoding, Baoding, Hebei 071000, P. R. China

**Keywords:** Minimally Invasive Extraction, Alveolar Ridge Preservation, Piezosurgery, Guided Bone Regeneration

## Abstract

**Objective:**

To compare the effects of high-speed turbodrill root extraction and piezosurgery tooth socket enlargement on the alveolar ridge preservation of maxillary anterior teeth.

**Methods:**

Thirty-six clinically eligible patients admitted to the No.2 Hospital of Baoding or the Baoding First Central Hospital from January 2018 to November 2019 were selected and randomly divided into two groups. Group-A were extracted by high-speed turbodrill root extraction, while Group-B were extracted by piezosurgery tooth socket enlargement. After extraction, GBR bone grafting and soft tissue transplantation were performed on the extraction sockets. The extraction time, integrity rate of labial bone plate of the extraction socket, pain-free rate, satisfaction rate, reduction of the height and width of the alveolar ridge, alveolar bone mineral density score, and new bone contour score of the alveolar bone of two groups were compared.

**Result:**

Group-B was significantly superior to Group-A in terms of tooth extraction time, pain-free rate, satisfaction rate and reduction of alveolar ridge height at three sites on the palatal side, with a statistically significant difference (*p<*0.05).

**Conclusions:**

Piezosurgery tooth socket enlargement is more worthy of clinical application due to its advantages of less impact on the preservation of the palatal alveolar ridge height of the maxillary anterior teeth, shorter tooth extraction time, postoperative pain-free rate and high final satisfaction rate.

## INTRODUCTION

With the rapid development of implant prosthodontics, an increasing number of patients choose to undergo implant restoration. Soft and hard tissue support is essential for the sustaining success of implant restoration. In order to obtain adequate bone support and favourable soft tissue closure, soft and hard tissue augmentation is often required in clinical practice. Immediate intervention during tooth extraction can maximize the protection of the alveolar bone at the implant site after tooth extraction, which is of great significance to the restoration of implant dentures. Furthermore, subsequent soft and hard tissue augmentation treatments can be dispensed with.^1^

Relevant studies have confirmed that the damage to the soft and hard tissues during tooth extraction will affect the postoperative wound healing. During tooth extraction, the damage to the surrounding soft and hard tissues should be minimized, and the soft and hard tissues around the affected tooth should be protected to the maximum extent to prevent bone absorption caused by trauma. To this end, priority is given to the search for effective and minimally invasive tooth extraction techniques by domestic and foreign scholars in their research.^2^ In this study, the effects of two minimally invasive tooth extraction techniques on the alveolar ridge preservation of maxillary anterior teeth were compared for reference in clinical application.

## METHODS

A total of 36 patients admitted to The No.2 Hospital of Baoding and Baoding First Central Hospital from January 2018 to November 2019 were selected, all of whom were required to have affected teeth extracted. All the patients were randomly divided into Group-A and Group-B. The study was approved by the Institutional Ethics Committee of Baoding First Central Hospital on March 20, 2018 (No.[2018]028).

### Inclusion criteri

- Patients with no residual root of a single maxillary anterior tooth, no acute inflammation, no periodontitis or periapical lesions, no root fracture or bone adhesion, and no serious dental or periodontal diseases in adjacent teeth.
- Patients without systemic diseases and able to tolerate tooth extraction.
- Patients who agree to the extraction of affected teeth, understand and can bear the risks and complications of tooth extraction, do not require immediate implant repair, explain the plan in detail and sign informed consent.

### Exclusion criteria:

- Patients with uncontrolled systemic diseas.
- Severe defect of labial bone plate or soft tissue, and require immediate implantation.

Preoperative clinical examination was carried out carefully to judge the condition of affected tooth, root and periradicular bone. Periodontal membrane separator was utilized to separate gingival and periodontal memb

### Group-A (high-speed turbodrill root extraction):

A high-speed turbodrill was used to divide the root in the mesial-distal direction from the labial 1/3 of the residual root to below the apical 1/3, and the affected tooth was divided into the labial and lingual sides. Most of the labial parts were released by themselves and taken out with vascular forceps. In the periodontal gap on the palatal side of the affected tooth, a minimally invasive tooth extraction knife produced by HuFriedyGroup was used to cut off the periodontal ligament and enlarge the tooth socket by sticking to the root surface. An appropriate minimally invasive extraction forcep was selected to extract the affected teeth separately by rotation.

### Group-B (piezosurgery tooth socket enlargement):

The piezosurgery L2 working tip produced by SATELEC was inserted into the periradicular gap. The working tip was repeatedly and evenly pulled back and forth in the near, far and palatal direction along the root surface, and the target depth was reached at the turning point of the working tip. An appropriate minimally invasive extraction forcep was selected to extract the affected teeth separately by rotation. In both groups, the granulation tissue and residual periodontal membrane in the extraction socket were gently scraped, and the extraction socket was rinsed with normal saline.

The microporous titanium mesh was trimmed and bent into a saddle shape according to the size of the extraction wound. After tooth extraction, artificial bone powder was filled into the extraction socket, and then the microporous titanium mesh was covered and fixed.

### Soft tissue transplantation:

The palatal mucosa was cut with a soft tissue ring cutter, stripped and repaired along the edge of the ring incision, and planted on the surface of the extraction wound, which was fixed on the gingiva around the edge of the patient’s tooth socket via interrupted sutures.

### Clinical observation:

After tooth extraction, the integrity of the labial bone plate on the extracted tooth socket was recorded as complete, mild, moderate and severe damage, respectively. One week after the operation, the graft rejection, the infection of the tooth extraction wound and the healing of the transplanted soft tissue were observed. Patients’ postoperative pain was recorded, and the pain-free rate was calculated. Six months after the operation, patients’ satisfaction with the tooth extraction process and the final effect of alveolar crest retention was evaluated, and the satisfaction rate of each item was calculated respectively.

Immediately after tooth extraction and six months after tooth extraction, CBCT was taken under the same projection conditions. The apex of the palate or the lower edge of the mandible was selected as the fixed reference point to make the line S perpendicular to the long axis of the affected tooth. The distances from the mesial, midpoint and distal to S of labial alveolar ridge were denoted as Hb1, Hb2 and Hb3 of labial alveolar ridge, the distances from that of palatal alveolar ridge were denoted as Hp1, Hp2 and Hp3, and the distances between the mesial point, midpoint and distal point of labial-palatal alveolar ridge were denoted as alveolar ridge width W1, W2, W3. The alveolar bone density was scored by Zarb classification: two points:good bone mineral density; One point:moderate bone mineral density. Zero point:poor bone mineral density. Score of new bone contour of alveolar bone: good new bone contour of the alveolar bone (two points); moderate new bone contour of the alveolar bone (one point); poor new bone contour of the alveolar bone (zero point).

### Statistical analysis:

All data in this study were analyzed by SPSS 23.0 statistical software. The measurement data conforming to normal distribution were expressed as mean ± S. T-test was used for comparison between the two groups, and χ^2^ test was used for comparison of count data. P<0.05 indicates a statistically significant difference.

## RESULTS

No transplant rejection, no infection of the tooth extraction wound and normal healing of transplanted soft tissue were found. As shown in Table-I, patients in Group-B had mild postoperative pain and higher satisfaction, with a statistically significant difference (p<0.05). The average extraction time of Group-A was significantly longer than that of Group-B (p<0.05). Table-I. Group-B was significantly better than Group-A in the reduction of alveolar ridge height at three sites on the palatal side, with a statistically significant difference (p<0.05) Table-II.

**Table-I:** Comparison of tooth extraction time,e integrity rate of labial bone plate of extraction socket, pain-free rate and satisfaction rate between the two groups.

Group	Number of cases	Tooth extraction time (min)	Integrity rate of labial bone plate of extraction socket	Pain-free rate	Satisfaction rate
Group-A	18	3.88±1.04	97.3	67.0	74.5
Group-B	18	2.31±0.88	98.5	81.0	97.0
t/χ^2^		t=4.89	χ^2^=15.5	χ^2^=10.30	χ^2^=18.61
P		<0.001	0.603	0.041	0.018

**Table-II:** Comparison of height and width reduction values, alveolar bone mineral density score, and new bone contour score of alveolar bone between the two groups

Group	No of cases	Reduction value in labial alveolar ridge height h/mm			Reduction value of palatal alveolar ridge height h/mm			Reduction value of alveolar ridge width h/mm			Alveolar bone density score/point	New bone contour score of alveolar bone/point

		Mesial Hb1	Midpoint Hb2	Distal Hb3	Mesial Hp1	Midpoint Hp2	Distal Hp3	Mesial W1	Midpoint W2	Distal W3		
Group-A	18	1.06±0.34	1.23±0.62	1.08±0.56	0.73±0.21	0.87±0.19	0.59±0.32	0.53±0.22	0.55±0.23	0.51±0.21	1.93±0.26	1.94±0.31
Group-B	18	1.05±0.43	1.14±0.57	1.04±0.56	0.60±0.16	0.71±0.21	0.40±0.21	0.55±0.24	0.58±0.20	0.56±0.18	1.96±0.18	1.93±0.28
t		0.08	0.45	0.21	2.09	2.40	2.11	0.26	0.42	0.77	0.40	0.10
P		0.94	0.65	0.83	0.04	0.02	0.04	0.80	0.68	0.45	0.69	0.92

## DISCUSSION

For the sustaining success of implant restoration and the ideal aesthetic restoration effect, a variety of methods have been adopted by researchers to preserve the alveolar bone and its surrounding soft tissue. As per Tan et al systematic review in early 2004, Hämmerle et al.^3^,^4^ studied the changes of tooth socket extraction after implanting various biomaterials such as autologous bone, allogeneic bone, xenogeneic bone and artificial synthetic bone with different barrier membranes. They named the technique alveolar ridge preservation. With the continuous development of site preservation techniques in recent years, the generalized site preservation techniques include GBR bone grafting, minimally invasive tooth extraction and implantation of extraction sockets (including immediate implantation and early implantation).^5^ Among them, minimally invasive tooth extraction is the first step of bone preservation, including minimally invasive concept, minimally invasive instruments and minimally invasive methods.^6^ In this study, the effect of minimally invasive tooth extraction technique on GBR bone grafting was firstly investigated, and its effect on extraction socket implantation and GBR bone grafting combined with simultaneous extraction socket implantation will be further studied in the future. In this way, better clinical guidance on minimally invasive tooth extraction technique can be practiced.

Studies have shown that the residual alveolar ridge presents irreversible bone absorption after tooth extraction.^7^-^9^ Almost 70%-80% of the total alveolar bone resorption occurs within three months of tooth extraction, at which time the width and height of the alveolar ridge decrease on average by (2.6-4.6) mm and (0.4-3.9) mm, respectively, with the most distinct absorption in the labial and buccal bone plates.^10^,^11^

In traditional tooth extraction, it is inevitable that the soft and hard tissues around the affected tooth will be damaged.^12^,^13^ In particular, the labial bone plate in the anterior teeth area is weak, and tooth extraction often leads to the destruction of the labial bone plate.^14^-^17^ Therefore, it is of great significance for the retention of the labial bone plate in the maxillary anterior region. This is also the reason why the labial bone plate integrity rate was selected as one of the evaluation indicators in this study. How to keep the bone wall of alveolar socket intact, especially the bone at the top of alveolar socket, minimize the absorption of alveolar bone after tooth extraction, and achieve an accurate and predictable tooth extraction treatment are still the main problems to be solved. Among them, reducing trauma during tooth extraction is an indispensable link.^18^

Root extraction is often used for the extraction of multiple posterior teeth, by which multiple teeth are separated into 2-3 independent single tooth units and removed one by one, boasting the advantages of time saving, high efficiency and less trauma.^19^ Consequently, in Group-A, 1/3 of the labial root was removed using the root splitting technique first, and then the residual root was removed with the help of a minimally invasive extraction knife without extrusion pressure on the labial bone plate. Piezosurgery has the advantage of inserting flat, thin and multi-angle working points into the periradicular gap using the principle of ultrasonic oscillation to achieve the cutting and bone removal in the alveolar cavity, which neither produces mechanical extrusion nor rotational grinding, and is minimally invasive, precise and predictable.^20^

In contrast, Group-B selected appropriate minimally invasive dental forceps to clamp out the residual root after the gap augmentation with piezosurgery. As shown in this study, piezosurgery was carried out to reduce the reduction of palatal bone plate in the extractive socket. The reason is that the thin and flat working tip can be inserted into the periradicular gap to allow bidirectional cutting of the medial alveolar plate and part of the bone, which preserves the bone at the top of the alveolar ridge without microfracture. Both extraction methods showed satisfactory protection of the labial bone plate. Therefore, the height of the alveolar ridge on the labial side was not significantly reduced, and the comparison between the two was not statistically significant.

### Limitations of this study:

The number of subjects included in this study was limited, so the conclusions drawn may not be very convincing. In addition, no significant difference was found in the preservation of the width of the alveolar bone, which may be due to the fact that the piezosurgery operations were confined to the periodontal gap and there was no large displacement in the horizontal space.

## CONCLUSION

Both minimally invasive tooth extraction methods show preferable preservation effect on the maxillary anterior teeth, well preserve the alveolar bone and reduce bone trauma. However, piezosurgery tooth socket enlargement is more worthy of clinical application due to its advantages of less impact on the preservation of the palatal alveolar ridge height of the maxillary anterior teeth, shorter tooth extraction time, postoperative pain-free rate and high final satisfaction rate.

### Authors’ Contributions:

**SJ & PW:** Designed this study,prepared the manuscript, are responsible and accountable for the accuracy and integrity of the work.

**BZ & ZL:** Collected and analyzed clinical data.

**JG:** Data analysis, Significantly revised this manuscript.
